# Measurement of changes in uterine and fibroid volume during treatment of heavy menstrual bleeding (HMB)

**DOI:** 10.1093/hropen/hoad021

**Published:** 2023-05-22

**Authors:** K Yin, L Whitaker, E Hojo, S McLenachan, J Walker, G McKillop, C Stubbs, L Priest, M Cruz, N Roberts, H Critchley

**Affiliations:** Edinburgh Imaging Facility QMRI, University of Edinburgh, Edinburgh, UK; MRC Centre for Reproductive Health, University of Edinburgh, Edinburgh, UK; MRC Centre for Reproductive Health, University of Edinburgh, Edinburgh, UK; Royal Infirmary of Edinburgh, NHS Lothian, Edinburgh, UK; Royal Infirmary of Edinburgh, NHS Lothian, Edinburgh, UK; Royal Infirmary of Edinburgh, NHS Lothian, Edinburgh, UK; Birmingham Clinical Trials Unit, University of Birmingham, Birmingham, UK; Birmingham Clinical Trials Unit, University of Birmingham, Birmingham, UK; Departamento de Matemáticas Estadística y Computación, University of Cantabria, Santander, Spain; Edinburgh Imaging Facility QMRI, University of Edinburgh, Edinburgh, UK; MRC Centre for Reproductive Health, University of Edinburgh, Edinburgh, UK; MRC Centre for Reproductive Health, University of Edinburgh, Edinburgh, UK

**Keywords:** uterus, imaging, fibroids, adenomyosis, leiomyoma, progesterone

## Abstract

**STUDY QUESTION:**

Does application of an unbiased method for analysis of magnetic resonance (MR) images reveal any effect on uterine or fibroid volume from treatment of heavy menstrual bleeding (HMB) with three 12-week courses of the selective progesterone receptor modulator ulipristal acetate (SPRM-UPA)?

**SUMMARY ANSWER:**

Application of an unbiased method for analysis of MR images showed that treatment of HMB with SPRM-UPA was not associated with a significant reduction in the volume of the uterus or in the volume of uterine fibroids.

**WHAT IS KNOWN ALREADY:**

SPRM-UPA shows therapeutic efficacy for treating HMB. However, the mechanism of action (MoA) is not well understood and there have been mixed reports, using potentially biased methodology, regarding whether SPRM-UPA has an effect on the volume of the uterus and fibroids.

**STUDY DESIGN, SIZE, DURATION:**

In a prospective clinical study (with no comparator), 19 women with HMB were treated over a period of 12 months with SPRM-UPA and uterine and fibroid size were assessed with high resolution structural MRI and stereology.

**PARTICIPANTS/MATERIALS, SETTING, METHODS:**

A cohort of 19 women aged 38–52 years (8 with and 11 without fibroids) were treated with three 12-week courses of 5 mg SPRM-UPA given daily, with four weeks off medication in-between treatment courses. Unbiased estimates of the volume of uterus and total volume of fibroids were obtained at baseline, and after 6 and 12 months of treatment, by using the Cavalieri method of modern design-based stereology in combination with magnetic resonance imaging (MRI).

**MAIN RESULTS AND THE ROLE OF CHANCE:**

Bland–Altman plots showed good intra-rater repeatability and good inter-rater reproducibility for measurement of the volume of both fibroids and the uterus. For the total patient cohort, two-way ANOVA did not show a significant reduction in the volume of the uterus after two or three treatment courses of SPRM-UPA (*P* = 0.51), which was also the case when the groups of women with and without fibroids were considered separately (*P* = 0.63). One-way ANOVA did not show a significant reduction in total fibroid volume in the eight patients with fibroids (*P* = 0.17).

**LIMITATIONS, REASONS FOR CAUTION:**

The study has been performed in a relatively small cohort of women and simulations that have subsequently been performed using the acquired data have shown that for three time points and a group size of up to 50, with alpha (Type I Error) and beta (Type II Error) set to 95% significance and 80% power, respectively, at least 35 patients would need to be recruited in order for the null hypothesis (that there is no significant reduction in total fibroid volume) to be potentially rejected.

**WIDER IMPLICATIONS OF THE FINDINGS:**

The imaging protocol that we have developed represents a generic paradigm for measuring the volume of the uterus and uterine fibroids that can be readily incorporated in future studies of medical treatments of HMB. In the present study, SPRM-UPA failed to produce a significant reduction in the volume of the uterus or the total volume of fibroids (which were present in approximately half of the patients) after either two or three 12-week courses of treatment. This finding represents a new insight in respect of the management of HMB using treatment strategies that target hormone-dependence.

**STUDY FUNDING/COMPETING INTEREST(S):**

The UPA Versus Conventional Management of HMB (UCON) trial was funded by the EME Programme (Medical Research Council (MRC) and National Institutes of Health Research (NIHR)) (12/206/52). The views expressed in this publication are those of the authors and not necessarily those of the Medical Research Council, National Institute for Health Research, or Department of Health and Social Care.

Medical Research Council (MRC) Centre grants to the Centre for Reproductive Health (CRH) (G1002033 and MR/N022556/1) are also gratefully acknowledged. H.C. has clinical research support for laboratory consumables and staff from Bayer AG and provides consultancy advice (All paid to Institution) for Bayer AG, PregLem SA, Gedeon Richter, Vifor Pharma UK Ltd, AbbVie Inc., and Myovant Sciences GmbH. H.C. has received royalties from UpToDate for an article on abnormal uterine bleeding. L.W. has received grant funding from Roche Diagnostics (Paid to Institution). All other authors have no conflicts to declare.

**TRIAL REGISTRATION NUMBER:**

The study reported here is an embedded mechanism of action study (no comparator) within the UCON clinical trial (registration ISRCTN: 20426843).

WHAT DOES THIS MEAN FOR PATIENTS? This is the first time that a mathematically rigorous and unbiased methodology has been applied in a clinical study of the effect of treatment of heavy menstrual bleeding (HMB) on the structure of the uterus. This has shown that in women with and without fibroids, three 12-week courses of the selective progesterone receptor modulator ulipristal acetate (SPRM-UPA) did not, on average, produce a significant reduction in the volume of the uterus, whether or not fibroids were present, or in the total volume of fibroids.

## Introduction

Heavy menstrual bleeding (HMB) is menstrual blood loss which interferes with a woman’s physical, social, emotional and/or material quality of life (NICE, 2018). Since it has a community prevalence of at least 25% and is a significant burden to healthcare systems (Shapley *et al.*, 2004; Whitaker and Critchley, 2016), providing effective treatment remains a clinical area of unmet need (RCOG, 2014; NICE, 2018; Geary *et al.*, 2019; Critchley *et al.*, 2020a). Existing medical treatments for HMB are often ineffective or associated with unacceptable side effects (Sweet *et al.*, 2012). However, surgical interventions (e.g. hysterectomy) are not appropriate for many women, especially given the trend toward later births (Myrskyla *et al.*, 2017; Inter Lace Study Team 2019) and the need to preserve fertility. There is therefore a requirement to develop safe, simple and acceptable, fertility sparing medical treatments for HMB.

Previously, in this laboratory, we have developed a novel measurement protocol for obtaining unbiased estimates of the volume of the uterus and uterine fibroids. The method is unbiased by mathematical design and is efficient and highly precise to apply (Thrippleton *et al.*, 2015). In the present study, the first clinical application of this protocol to study the effect of a medical treatment for the symptom of HMB is reported. The study was made possible by the embedding of a mechanism of action (MoA) investigation (EME Programme; EME 12/206/52) within the MRC/NIHR-funded UCON clinical trial (EudraCT 2014-003408-65; REC 14/LO/1602) to investigate the efficacy of treatment of HMB with ulipristal acetate (UPA), which is a selective progesterone receptor modulator (SPRM), in a cohort comprising similar sized groups of women with and without fibroids.

The reason that SPRMs may provide a solution for treating HMB comes from the mounting evidence that progesterone, and the progesterone receptor (PR), play a pivotal role in both menstruation and in the growth and development of uterine fibroids (Bulun, 2013; Maybin and Critchley, 2016; Wagenfeld *et al.*, 2016). Uterine fibroids, often referred to as leiomyomas, are benign tumours of uterine muscle found in up to 80% of women of reproductive age. Although they do not always cause symptoms, they are one of nine potential causes of HMB named in the PALM-COEIN acronym, comprising: polyps, adenomyosis, leiomyoma, malignancy, coagulopathy, ovulatory disorders, endometrial, iatrogenic, not otherwise classified (Baird *et al.*, 2003; Munro *et al.*, 2011, 2018). The SPRM ulipristal acetate (UPA) has demonstrated control of HMB in over 90% of women, and amenorrhoea in over 70% of women, in the PEARL clinical trials (Donnez *et al.*, 2012a,b, 2014, 2016). In these and other studies (Levens *et al.*, 2008; Nieman *et al.*, 2011), UPA was shown to be efficient and safe in reducing average uterine fibroid volume in both the short and long term (Donnez *et al.*, 2018). However, it has also been reported that not all patients respond to UPA treatment (Woodhead *et al*., 2018) and the extent of the response may depend on whether fibroids are present as well as their number, location, and size (Yun *et al*., 2018; Netter *et al*., 2019).

The state of the art protocol that we have used in this study uses a combination of high resolution magnetic resonance imaging (MRI) and modern design based stereology (Roberts *et al.*, 2000). The Cavalieri method of modern stereology is unbiased by design and has predictable precision. When used in combination with MRI for measuring the volume of the uterus and fibroids, it has been previously shown (in our laboratory) to provide excellent repeatability and reproducibility and is very efficient to apply (Thrippleton *et al.*, 2015).

The main objective of the present study was to apply the Cavalieri method in combination with MRI to measure changes in the volume of the uterus in women with and without fibroids, before, during, and after receiving three 12-week courses of treatment with UPA. By additionally measuring the total volume of fibroids in the group of women with fibroids, it was possible to pursue a secondary aim of investigating whether potential changes in the volume of the uterus are influenced by the presence of fibroids.

## Materials and methods

### Patients

A cohort of 19 women, aged 38–52 years (median 44; first quartile 42; third quartile 47) with HMB, participated in an embedded mechanism of action (MoA) study within the MRC/NIHR (EME Programme; EME 12/206/52) UCON clinical trial (registration ISRCTN: 20426843). After recruitment, the cohort was screened using transvaginal and/or abdominal ultrasound. This was to exclude patients with pathologies such as uterine congenital malformation and to exclude patients with large fibroids. In particular, if a patient had fibroids, they were not included if the size of the uterus was greater than that predicted at 14 gestational weeks, which corresponds to 14 cm, if the uterine cavity length was >11 cm, or if the diameter of a fibroid with a submucosal component was greater than 2 cm. Other exclusion criteria related to contraindications for the use of UPA or the levonorgestrel-releasing intrauterine system. Otherwise, the participant cohort comprised two similar-sized groups of women, with and without uterine fibroids, in whom adenomyosis might also occasionally be present. Demographic information for participants including age, body mass index (BMI), ethnicity, and parity is presented in Table 1. Each participant was administered three courses of UPA at a dose of 5 mg orally once daily for 12 weeks, with four weeks off medication in between treatment courses. High resolution structural MRI (see below) was performed before (i.e. baseline MRI), after 6 months of UPA treatment (i.e. in the final week of the second course of medication: mean number of courses ± standard deviation (SD) 2.00 ± 0.16 courses; range from 1.60 to 2.52 courses), and again after 12 months of UPA treatment (i.e. in the final week of the third course of medication: mean ± SD 2.96 ± 0.09 courses; range from 2.66 to 3.00 courses).

**Table 1.** Demographic information.

	Fibroids present	Fibroids absent	All	*P*-value
	(n = 8)	(n = 11)	(n = 19)	
**Age^1^ [mean (range)]**	46.1 (42–51)	42.5 (38–52)	44 (38–52)	0.04
**BMI^2^ [kg/m^2^, (range)]**	26.1 (21.9–35.2)	33.5 (23.9–48.9)	30.4 (21.9–48.9)	0.03
**Ethnicity [n (%)]**				
** British White**	7 (88%)	10 (91%)	17 (89%)	
** Southern European**	1 (13%)	0 (0%)	1 (5%)	
** Black**	0 (0%)	0 (0%)	0 (0%)	
** Chinese**	0 (0%)	0 (0%)	0 (0%)	
** Indian**	0 (0%)	0 (0%)	0 (0%)	
** Other Asian**	0 (0%)	0 (0%)	0 (0%)	
** Mixed**	0 (0%)	1 (9%)	1 (5%)	
**Parity [n (%)]**				
** Nulliparous**	2 (25%)	1 (9%)	3 (16%)	
** 1**	2 (25%)	1 (9%)	3 (16%)	
** 2**	3 (38%)	2 (18%)	5 (26%)	
** 3**	1 (13%)	5 (45%)	6 (32%)	
** ≥4**	0 (0%)	2 (18%)	2 (11%)	
**Adenomyosis^3^ [n (%)]**				0.99
** Present**	2 (25%)	4 (36%)	6 (32%)	
** Absent**	6 (75%)	7 (64%)	13 (68%)	

1 Age normally distributed—unpaired *t* test.

2 BMI not normally distributed—Mann Whitney.

3 Adenomyosis—Fisher’s exact test.

### Magnetic resonance imaging

MRI investigations were performed on a 3T Verio system (Siemens Healthineers, Erlangen, Germany) installed in the research setting of the Edinburgh Imaging facility at The Queen’s Medical Research Institute (QMRI), University of Edinburgh, in a manner consistent with the recommendations of the Safety Committee of the International Society of Magnetic Resonance in Medicine (ISMRM) (Calamante *et al.*, 2015). On each occasion, contiguous series of T2-weighted (T2W) MRI images were acquired in the sagittal plane using a fast spin echo (FSE) pulse sequence with the following acquisition parameters, repetition time (TR): 3950 ms, echo time (TE): 100 ms, flip angle: 150°, slice thickness: 5 mm, spacing between slices: 5 mm, field of view (FOV): 199 × 199 mm, matrix size: 384 × 288, and one average. The FSE T2W MR images were reviewed together with standard diagnostic series of MR images by a radiologist who also noted whether there were imaging signs to indicate the presence of adenomyosis.

### Stereology

Volume estimates were obtained using the Cavalieri method of modern design stereology in combination with point counting on the T2W FSE MR images using protocols that we have developed, and which were described in detail in a previous publication (Thrippleton *et al.*, 2015). Firstly, for all patients, estimates of the volume of the body of the uterus between the fundus and the internal os (i.e. not including the cervix) were obtained. Secondly, for patients with fibroids, total fibroid volume was estimated on the same images. For patients with fibroids, the volume of the body of the uterus not including fibroids was obtained by subtracting total fibroid volume.

To obtain the volume estimates, the Cavalieri method was applied using EasyMeasure software (Puddephat, 1998). The distance between test points in the square grid was set to between 7.77 and 10.36 mm depending on the size of the uterus (i.e. grid size in EasyMeasure was set to between 15 and 20 pixels). The predicted coefficient of error (CE) was also computed for each volume estimate by using well established mathematical formulae (Matheron, 1971; Kiêu, 1997; Gundersen *et al.*, 1999; Kiêu *et al.*, 1999). After approximately 80% of the study had been completed, an intra-rater repeatability study was undertaken in which a radiologist (S.M.) performed repeat measurements on two occasions, and an inter-rater reproducibility study was performed for two observers (one radiologist (S.M.) and one medical imaging student researcher (K.Y.)) who independently obtained volume estimates for the uterus and the volume of the three largest fibroids on the MR images. These studies were performed for the three largest fibroids as it was convenient to mark their location and to be sure that the observers were investigating the same fibroid at the three different time points of the study.

### Statistical analysis

Statistical analysis was performed using R software (R Core Team 2018). For the intra-rater repeatability and inter-rater reproducibility studies, agreement was assessed by Bland–Altman analysis (Gerke, 2020). The next analyses, including a two-way repeated measures ANOVA and a one-way repeated measures ANOVA, were performed using the ezANOVA function in R (further information can be found at https://www.rdocumentation.org/packages/ez/versions/4.4-0/topics/ezANOVA).

The two-way repeated measures ANOVA was performed to test the null hypotheses that the population mean of the volume of the uterus in the total patient cohort is the same at all time points, and that there is no significant difference in changes in volume between the two groups, in comparison to the alternative hypotheses that the population mean volume of the uterus is significantly different at one or more time points, and that there is a significant difference in changes in volume between the two groups. In addition, a one-way repeated measures ANOVA was performed to test the null hypothesis that the population mean of the total volume of fibroids, in the group of eight patients in whom they were present, is the same at all time points in comparison to the alternative hypothesis that the population mean is significantly different at one or more time points. For both analyses, results were considered significant if *P* < 0.05. Finally, simulations were performed to establish the size of the patient groups that should be recruited for future studies in order to obtain specific levels of statistical significance in testing the above null hypotheses. The approach that was used is that proposed by (Kerns, 2012).

### Ethical approval

Approval for the study was obtained from the Lothian Research Ethics Committee (REC14/LO/1602), consent to participate was sought by a Gynaecologist, and written confirmation was kept by each participant and recruiting hospital and the UCON Trial Office. Patients were recruited between June 2015 and March 2020. The study was conducted in accordance with the principles of good clinical practice (GCP).

## Results

Analysis of the demographic information presented in Table 1 revealed that the patients with fibroids were significantly older and had a significantly lower BMI than the patients without fibroids. There were no significant differences in ethnicity or parity between the two groups, and also no significant difference with respect to the presence of adenomyosis, which was reported to be present in 2/8 (25%) of the patients with fibroids and in 4/11 (36%) of the patients without fibroids.

### Intra-rater repeatability and inter-rater reproducibility

**Figure 1.**
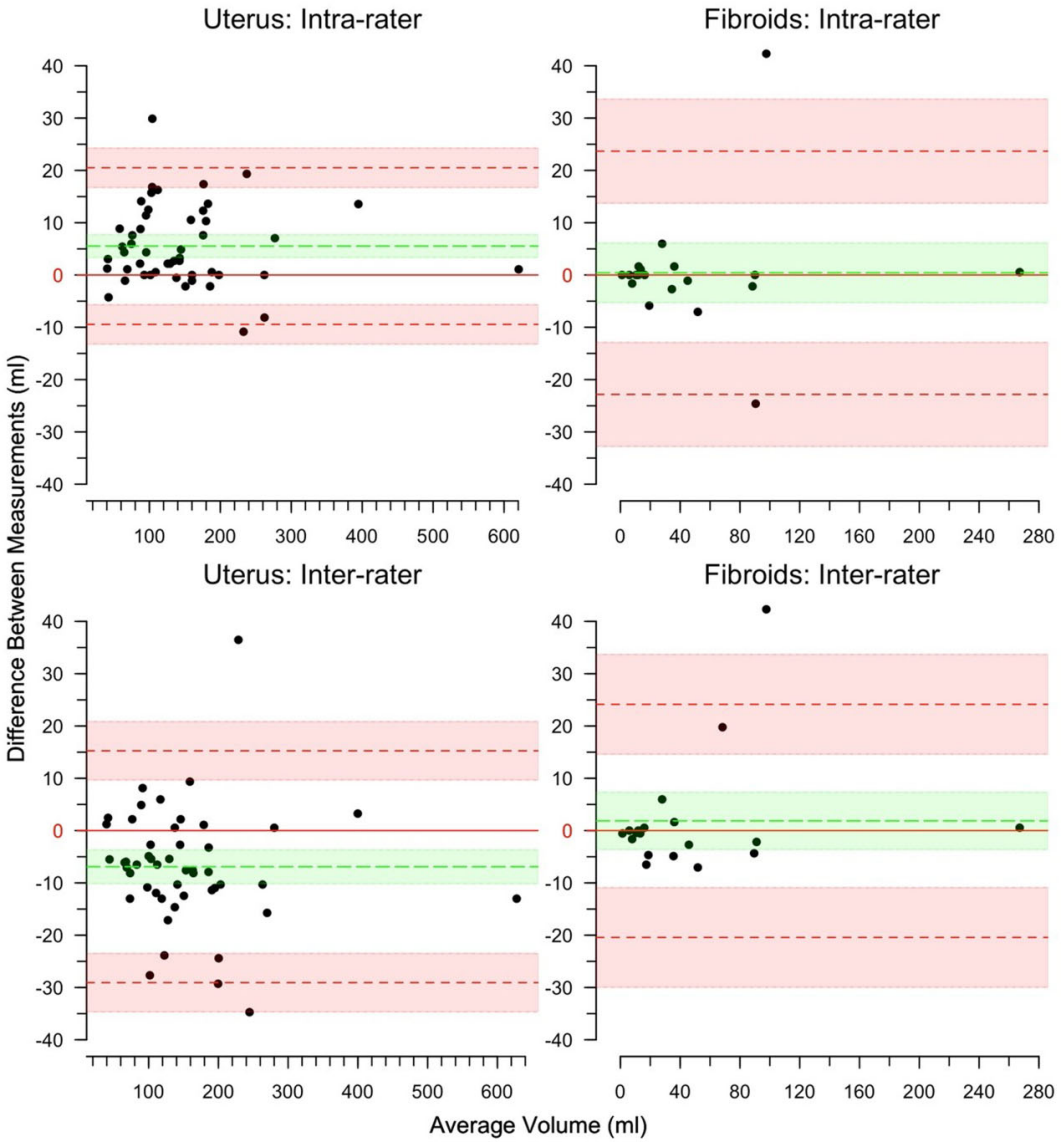
**Bland–Altman plot for repeatability and reproducibility of uterine and fibroid volume.** Results of intra-rater (top row) and inter-rater (bottom row) studies performed to determine repeatability and reproducibility for estimating the volume of the body of the uterus (left column) and of the three largest fibroids (right column). The dotted red lines within the light red area and the dotted green line within the light green area indicate limits of agreement (LoA) and Bias with 95% CI, respectively. The solid red horizontal line corresponds to no mean difference between the two measurements.

The average predicted CE for measurement of total fibroid volume was 3.3% (SD 4.7%, range 0.8% to 23.9%) and the average predicted CE for measurement of the volume of the body of the uterus was 1.4% (SD 0.7%, range 0.4% to 4.0%). The Bland–Altman plots in Fig. 1 show good intra-rater repeatability and good inter-rater reproducibility for measurement of both the volume of the three largest fibroids (i.e. analysis of 19 MR images referring to 8 patients at one or more of three time points) and the volume of the uterine body (i.e. analysis of 49 MR images referring to 19 patients at one or more of three time points). Values of the limits of agreement (LoA) and Bias computed for all four analyses are shown in Table 2. In the case of the three largest fibroids, the mean difference between repeat measures obtained by the same observer or by two different observers were within the boundary of the 95% confidence intervals (i.e. the region shaded light green in Fig. 1) and no significant bias was found. The corresponding data for the uterine body revealed a significant bias (*P* < 0.05) in both intra-rater (5.5 ml (95% CI 3.3 to 7.7 ml)) repeatability and inter-rater (6.9 ml (95% CI −3.7 to −10.2 ml)) reproducibility. However, in both cases the bias was small, being of the order 5%, and close to the predicted CE of the volume estimates.

**Table 2.** Bland–Altman analysis for intra-rater repeatability and inter-rater reproducibility studies.

	Intra-rater		Inter-rater	
	Uterine body	Uterine fibroids	Uterine body	Uterine fibroids
Bias (95% CI)	5.53* (3.34, 7.72)	0.42 (−5.3, 6.14)	−6.9* (−10.15, −3.65)	1.86 (−3.62, 7.34)
Limits of agreement (95% CI)				
Lower	−9.45 (−13.23, −5.67)	−22.83 (−32.78, −12.88)	−29.06 (−34.65, −23.47)	−20.43 (−29.96, −10.90)
Upper	20.5 (16.72, 24.28)	23.68 (13.73, 33.63)	15.26 (9.67, 20.85)	24.14 (14.61, 33.67)

* *P* < 0.05.

### Change in the volume of the uterus

**Figure 2.**
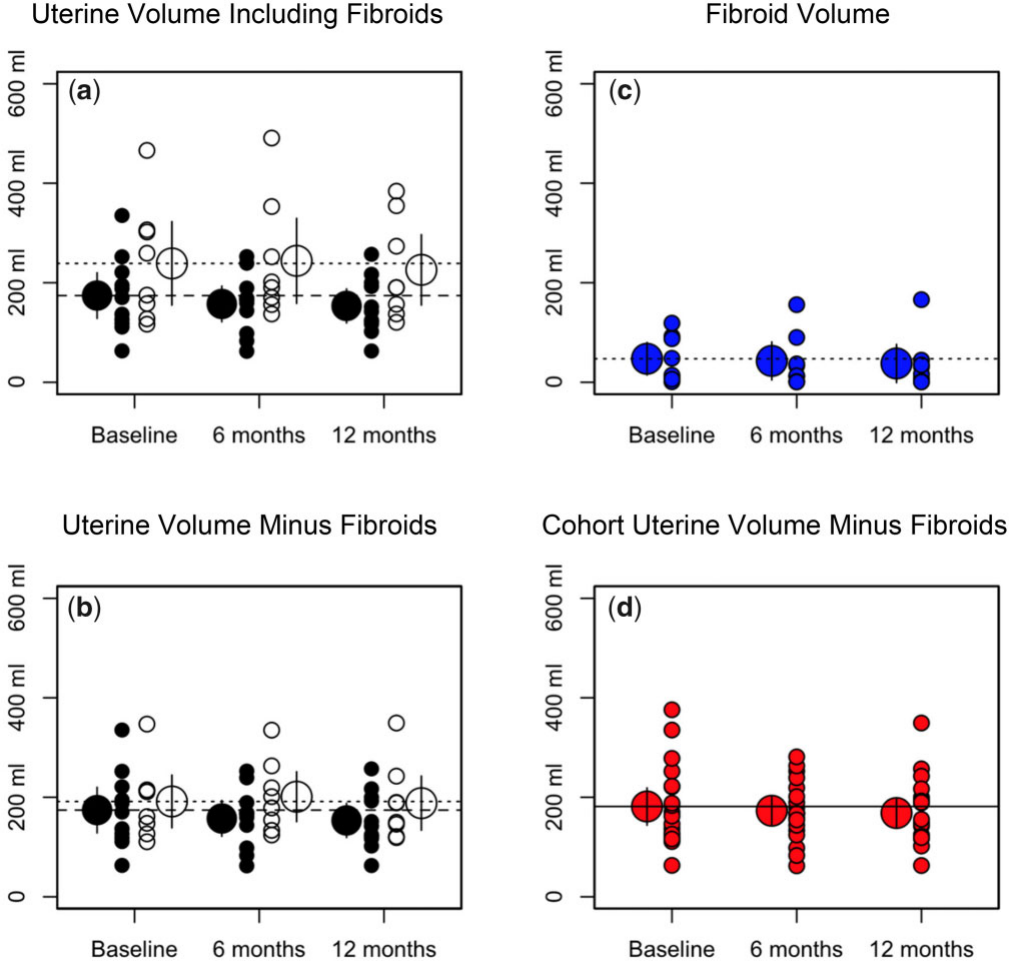
**Total uterine and fibroid volumes.** Individual data points (small circles) and mean values (large circles) of the volume of the uterus (**a**) with and (**b**) without the inclusion of total volume of fibroids are plotted at baseline and after 6 and 12 months of treatment with SPRM-UPA. Open circles refer to patients with fibroids and closed circles to patients without fibroids. Corresponding values are plotted in (**c**) for total fibroid volume in the group of patients with fibroids and in (**d**) for the volume of the uterus, excluding the volume of fibroids when present, in the combined cohort of patients.

The total volume of the uterus plus fibroids in the group of 8 patients in whom fibroids were present, and of the uterus in the group of 11 patients without fibroids, are plotted as open and closed symbols, respectively, at baseline and after two and three 12-week courses of treatment with SPRM-UPA in Fig. 2a. The same data are plotted in Fig. 2b, except that for the patients with fibroids, total fibroid volume is subtracted from the volume of the uterus. The total volume of the fibroids is plotted in Fig. 2c and the volume of the uterus, excluding the total volume of fibroids when present, is plotted for the combined cohort of all 19 patients in Fig. 2d. Application of the Shapiro test again indicated that the measures of the volume of the uterus obtained at the three time points were not normally distributed. Accordingly, the volumes were converted to logarithms, after which the same test confirmed that the resulting data were normally distributed, and sphericity of the data was confirmed by application of Mauchly’s test. Subsequent application of the two-way ANOVA confirmed the null hypotheses that that there was no significant change in the volume of the uterus in the total patient cohort (*P* = 0.51), and no significant difference between the two groups (*P* = 0.63), after two or three courses of treatment with the SPRM (UPA) (*P* > 0.05). There is no evidence that normal uterine tissue responds to UPA.

### Change in the total volume of fibroids

The total volume of uterine fibroids in the group of 8 patients in whom fibroids are present are plotted at baseline and after two and three 12-week courses of treatment with SPRM-UPA in Fig. 2c. Application of the Shapiro test again indicated that the volumes obtained at the three time points were not normally distributed. Accordingly, the fibroid volumes were converted to logarithms, after which the same test confirmed that the resulting data were normally distributed, and sphericity of the data was confirmed by application of Mauchly’s test. Subsequent application of the one-way repeated measures ANOVA confirmed the null hypothesis that there was no significant change in the total volume of fibroids in the group of 8 patients in whom they were present, either after two or three courses of treatment with the SPRM (UPA) (*P* = 0.17).

### Power calculations to assist in the design of future studies

Simulations were performed using the approach proposed by Kerns (2012) to establish, from the data acquired, the number of subjects to be recruited in order for future studies to be appropriately powered to obtain particular levels of significance. As above, the volumes of the uterus and fibroids were first converted to logarithms and after performing 1000 simulations, for three time points and a group size of up to 50, with alpha (Type I Error) and beta (Type II Error) set to 0.05 and 0.2, respectively, it was found that a total of at least 35 patients would need to be recruited in order for the null hypothesis (that there is no significant reduction in total fibroid volume) to potentially be rejected.

## Discussion

A mechanism of action (MoA) study embedded in a clinical trial has produced new results concerning the effect on uterine volume of courses of a medical treatment for the symptom of HMB, that targets the sex steroid dependent regulation of menstruation and has a role in growth of uterine fibroids (Critchley *et al.*, 2020b). In 19 women with symptoms of HMB, approximately half of whom had fibroids present in the uterus, no significant reduction was observed in the average volume of the uterus after either two or three 12-week courses of treatment with a PR modulator (UPA), irrespective of whether fibroids were present. Furthermore, for the eight patients with fibroids, no significant change on average was observed in total fibroid volume after two or three courses of treatment. The patients with fibroids were significantly older and had lower BMIs compared to the patients without fibroids. The principal finding is that treatment with a PR modulator (UPA) produced no significant reduction in either the volume of the uterus or total fibroid volume. Whilst it would be novel and highly interesting to consider whether the presence of adenomyosis was a potential confounding factor thatcould have influenced whether treatment with SPRM-UPA is associated with changes in the volume of the uterus and/or volume of uterine fibroids, the relatively small incidence of adenomyosis (i.e. 2 of the 8 patients with fibroids and 4 of the 11 patients without fibroids) precludes this investigation in the present study. There was no significant difference between the groups of participants with and without fibroids with respect to radiological evidence of the presence of adenomyosis. There is a dearth of appropriately powered clinical trials specific to use of SPRM treatment in patients with adenomyosis. Investigation of a potential relationship between changes in the volume of the uterus and/or volume of uterine fibroids and bleeding control in patients with HMB must also wait until a study can be performed for a larger cohort of patients and in whom the presence of adenomyosis is well characterized.

### Changes in the volume of the uterus due to treatment that impacts the progesterone–PR pathway

Early imaging studies of uterine pathology were performed by studying hysterosalpingogram images and provided sensitivity and specificity of 50% and 82.5%, respectively, for identifying the combination of fibroids and endometrial polyps (Soares *et al.*, 2000). Since the approach uses X-rays, it is now rarely used and has been replaced by ultrasound which performs better. In particular, transvaginal ultrasound has 90% sensitivity and 86.7% specificity for detecting the same pathologies (Cicinelli *et al.*, 1995; Becker *et al.*, 2002) and sonohysterography offers further improvements (Soares *et al.*, 2000).

Detailed three-dimensional (3D) measurement is challenging using ultrasound, and MRI offers distinct advantages. In particular, although both techniques have high sensitivity and high specificity for diagnosing the presence of submucosal fibroids (Dueholm *et al.*, 2001), MRI, by allowing acquisition of a systematic series of sections at constant intervals along a chosen scanning direction, also allows convenient application of the Cavalieri method to efficiently obtain unbiased estimates of fibroid volume as well as the volume of the body of the uterus. The volume of the body of the uterus has previously been measured in studies of infertility, menstrual disorders, pelvic masses, and ambiguous genitalia (Kelsey *et al.*, 2016). However, there have been only a few studies of potential changes in the volume of the uterus in the treatment of HMB. For example, a reduction in uterine volume has been reported in patients with fibroids treated with UPA (Trefoux Bourdet *et al.*, 2015; Baggio *et al.*, 2018), although neither of the trials recruited patients who did not have fibroids. Furthermore, the planimetry method, in which feature boundaries are exhaustively outlined by hand on a slice-by-slice basis, and the Calliper method, used in the above studies, are both problematic. In particular, bias is inherent in the latter as the approach is not a proper design for 3D volume estimation and may arise in the former due to difficulties in using a cursor to accurately trace the boundary of fibroid transects on MR images. The planimetry method is also less efficient and may lack reproducibility. The Cavalieri method is mathematically unbiased. However, when applied in combination with MRI, bias may arise on account of different observers, or the same observer on different occasions, perceiving the boundary of the structure of interest to lie in a different position. The bias recorded in the present study for measurement of the body of the uterus, but not fibroids, accords with the findings of Thrippleton *et al.* (2015), and being of the order of 5% is similar to the Coefficient of Error (CE) that is predicted for the volume estimates obtained using manual stereological analysis. The entire analysis was performed by a radiologist highly experienced in reporting MRI investigations of the uterus.

### Changes in the volume of fibroids due to treatment that impacts the PR pathway

Performing studies using MRI has the advantage that the same fibroid can be readily identified and measured at different time points. Treatment with a PR modulator (UPA) has been reported to produce a decrease in the volume of uterine fibroids in the so-called PEARL clinical trials (Donnez *et al.*, 2012a,b, 2014, 2016) and other studies (Nieman *et al.*, 2011; Ferrero *et al.*, 2016). However, the finding of the present study that individual fibroids may decrease, increase, or maintain the same volume, and that the average volume of fibroids is unaffected by treatment with PR modulation (UPA) is not unexpected on account of the studies by Yun *et al.* (2018) and Netter *et al.* (2019) who investigated which factors may predict the response of specific fibroids. In a retrospective analysis of 152 women using ultrasound, Yun *et al.* (2018) observed that, although there was no effect of fibroid location or initial volume, a significant reduction in the average volume was more likely with a fewer number of fibroids. However, measurements were obtained using ultrasound in some patients and MRI in others and the analysis was performed per patient rather than per fibroid. Furthermore, the time interval was not reported. More recently, Netter *et al.* (2019) performed an MRI study of 53 women who received a daily 5 mg dose of UPA over 3 months and measured the volume of the three largest fibroids on MR images obtained on average 117 days apart during treatment. In almost half of the women (51.2%) in whom at least two fibroids were present, the Intra-class Correlation Coefficient (ICC) for their respective percentage reduction was statistically non-significant, indicating that if one of the fibroids underwent a reduction in volume, the likelihood was that the other grew during UPA treatment. The authors did, however, report evidence to suggest, similar to Yun *et al.* (2018), that a log-linear relationship exists between response to treatment and the initial number of fibroids, such that the overall reduction in fibroid volume was relatively greater when only a few fibroids were present. The additional reports by both Yun *et al.* (2018) and Netter *et al.* (2019) that UPA produces a greater reduction in the volume of large than small fibroids is relevant to the interpretation of findings in the present study. In particular, the exclusion of women with very large fibroids from the present study could explain why a significant treatment effect was not detected.

With regard to the location of fibroids, Netter *et al.* (2019) additionally reported that intra-mural fibroids had a statistically significant lower response to PR modulator administration. They (Netter *et al.*, 2019) concluded that fibroids have their own individual predictive factors for response to PR modulator (UPA) treatment and also that there may be little or no link between this response and patient characteristics such as age and BMI.

### Normal variation and non-treatment-based changes in fibroid volume

To assist in interpreting the results of the present study, it is helpful to review knowledge of what changes in fibroid volume may be expected to occur in the absence of any treatment. Consistent with the reduction that occurs in clinical symptoms at the time of the menopause (Ross *et al.*, 1986), and that post-menopausal fibroids tend not to be large (Cramer and Patel, 1990), DeWaay *et al.* (2002) reported observing six small fibroids spontaneously resolving in women approaching the menopause. The women enrolled in the present study were aged between 38 and 52 years and there may therefore be a tendency for fibroids to be naturally reducing in volume in these women. Nevertheless, they presented with clinical symptoms of HMB.

There have been several studies which shed light on the changes in fibroid volume that may be occurring in patients prior to treatment. In particular, Tsuda *et al.* (1998) recruited 70 patients aged from 30 to 57 years and measured both the volume of fibroids and blood flow characteristics of the main uterine and fibroid arteries by using ultrasound at 3-month intervals for one year. Arteries specifically related to the fibroid could be detected for 52 (51.5%) of 101 fibroids and there was an increase in the volume of 24 (i.e. 46.2%) of these fibroids, compared to in only three (i.e. 6.1%) of 49 fibroids where a fibroid artery was not present. Peddada *et al.* (2008) recruited 72 patients aged from 24 to 54 years, with the stipulation that the women had at least one fibroid greater than 5 cm in diameter, and measured the changes in fibroid volume that occurred naturally over a period of 12 months. Growth rates for the 262 fibroids varied widely and were not influenced by fibroid size, location, BMI, or parity. Interestingly, only 7% of fibroids showed a regression in volume of greater than 20% and in the same women, individual fibroids sometimes increased or decreased at different rates despite a uniform hormonal milieu. More recently, Baird *et al.* (2020) recruited 1693 African–American women, who they suggested may be expected to develop fibroids at least 10 years earlier on average than white women. The women were aged between 23 and 35 years. In the course of the 18 months of the study, fibroids appeared in 9.4% of the 1123 women in whom no fibroids were present at the start of the study. With regard to the changes that were observed in fibroid volume over the course of the study, interestingly, very small fibroids (i.e. <1 cm diameter) were found to be very dynamic, exhibiting rapid growth but also a high chance of disappearing, whereas larger fibroids (i.e. >2 cm diameter) typically grew slowly. This may further explain why excluding women with fibroids greater than 2 cm in diameter from the present study may have made the detection of an effect of SPRM-UPA on fibroid growth unlikely. In order to identify factors that may predict how a patient will respond to treatment in future studies, measurement of MRI characteristics may be combined with molecular analysis to determine whether fibroid number, size, or location are linked to the same gene expression profiles. However, the very high variability in terms of number, location, and total volume of fibroids, observed in the present and above-mentioned studies provides a significant challenge for recruiting cohorts of sufficient size (i.e. >35 patients) in order to have sufficient power to be able to detect significant effects.

In summary, employment of the unbiased Cavalieri method to analyze T2-weighted FSE MR images obtained in this embedded exploratory MoA study performed in a cohort of 19 women with HMB did not find evidence that the SPRM-UPA produced a significant reduction in the volume of the uterus, after either two or three 12-week courses of treatment. Similarly, there was no significant reduction in the total volume of fibroids which were present in approximately half of the patients. The protocol that we have developed represents a generic paradigm for measuring the volume of the uterus and uterine fibroids that can be readily incorporated in future studies of medical treatments of HMB, including recent strategies that target hormone dependence and assess uterine and fibroid size (Schlaff *et al.*, 2020).

## Data Availability

The data underlying this article will be shared upon reasonable request to the corresponding author.
